# Active ingredients are reported more often for pharmacologic than non-pharmacologic interventions: an illustrative review of reporting practices in titles and abstracts

**DOI:** 10.1186/1745-6215-14-146

**Published:** 2013-05-20

**Authors:** Nicola McCleary, Eilidh M Duncan, Fiona Stewart, Jill J Francis

**Affiliations:** 1 Aberdeen Health Psychology Group, Health Services Research Unit, University of Aberdeen, 1st Floor, Health Sciences Building, Foresterhill, Aberdeen, AB25 2ZD, UK; 2 Aberdeen Health Psychology Group, Health Services Research Unit, University of Aberdeen, 2nd Floor, Health Sciences Building, Foresterhill, Aberdeen, AB25 2ZD, UK; 3 Health Services Research Unit, University of Aberdeen, 2nd Floor, Health Sciences Building, Foresterhill, Aberdeen, AB25 2ZD, UK; 4 Aberdeen Health Psychology Group, Health Services Research Unit, University of Aberdeen, 3rd Floor, Health Sciences Building, Foresterhill, Aberdeen, AB25 2ZD, UK

**Keywords:** Active ingredients, Behaviour change interventions, Complex interventions, Illustrative review, Non-pharmacologic interventions, Pharmacologic interventions, Reporting standards

## Abstract

Key components of healthcare interventions include ‘active ingredients’ (intervention components that can be specifically linked to effects on outcomes such that, were they omitted, the intervention would be ineffective). These should be reported in titles and abstracts of published reports of randomized controlled trials (RCTs). However, reporting of non-pharmacologic interventions (NPIs), particularly behaviour change interventions (BCIs), is difficult, owing to their complexity. This illustrative review compares how pharmacologic interventions (PIs), NPIs and BCIs are specified in titles and abstracts to clarify how reporting of NPIs and BCIs can be improved. MEDLINE and Embase were searched for RCTs published in the *British Medical Journal*, *The Journal of the American Medical Association*, *The New England Journal of Medicine*, *The Lancet* and *Annals of Behavioral Medicine* from 2009 to March 2011. All types of intervention, participant and outcome were included. A random sample of 198 studies (sampled proportionally from included journals) stratified by intervention type (PI/NPI) was taken: 98 evaluated PIs, 96 evaluated NPIs and four evaluated both. Studies were coded for the presence or absence of key components. The frequency data were analyzed using the chi-square test. Active ingredients were named in 88% titles and 95% abstracts of PI reports, and in 51% titles and 71% abstracts of NPI reports, with a significant association between intervention type and reporting of active ingredients in titles (*χ*^2^(1) = 28.90; *P* < 0.001) and abstracts (*χ*^2^(1) = 16.94; *P* < 0.001). Active ingredients were named in BCI reports in 37% titles and 56% abstracts, and in other NPI reports in 66% titles and 86% abstracts. There was also a significant association between intervention type and reporting of active ingredients in titles (*χ*^2^(1) = 6.68; *P* = 0.010) and abstracts (*χ*^2^(1) = 8.66; *P* = 0.003). Reporting practices also differed for such components as the trial setting and intervention provider. This review highlights the need for improved reporting of NPIs (particularly BCIs) and indicates that a set of agreed labels and definitions for complex NPIs could facilitate standardized reporting. This would ensure that interventions can be faithfully replicated and that evidence for interventions can be appropriately synthesized.

## Background

Well designed and executed randomized controlled trials (RCTs) are the gold standard for assessing intervention efficacy and effectiveness, but study synthesis, comparison and reproducibility are compromised if interventions are poorly reported [1,2]. Articles should contain details of key study components (Table 1) including ‘active ingredients’ [1,3]. The term ‘active ingredient’ is frequently used to refer to the element within a pharmacologic intervention (PI) that is responsible for its therapeutic action. In contrast with PIs, non-pharmacologic interventions (NPIs) are usually complex, containing several interacting components that are all necessary for the intervention to be effective [4]. Such interventions may [5]:

•Involve several interacting components.

•Require many different behaviours from healthcare professionals or participants for successful delivery.

•Be aimed at different levels within an organization.

•Have many different types of outcome measurement.

•Be tailored to different contexts or settings within one study.

**Table 1:** **Checklists adapted from the CONSORT extension for abstracts**[1]**and the CONSORT extension for non-pharmacologic interventions**[2]

**Section of paper**	**Component to be reported**	**Details to be reported**
Title	Randomization	Specify the use of randomization
Abstract	Study design	Description of trial design
	Participants	Eligibility criteria and data collection setting
	Interventions	Interventions given to each group within the trial
	Objective	Objective or hypothesis of the trial
	Primary outcome	Specification of the primary outcome, result for each intervention group, estimated effect size and precision
	Randomization	Method used to allocate participants to intervention groups
	Blinding	Who was blinded to group assignment
	Numbers randomized and analyzed	Number of participants randomized to and analyzed in each intervention group
	Recruitment	Specify whether study is ongoing, closed to recruitment, or closed to follow-up
	Harms	Significant adverse events or side effects that occurred
	Conclusions	Evaluation of the results
	Trial registration	Trial register name and registration number
	Funding	Funding source
Abstract (non-pharmacologic interventions)	Full description of the experimental treatment; comparator; care providers administering intervention; centres within which intervention administered; trial blinding status	

CONSORT, Consolidated Standards of Reporting Trials.

The term ‘active ingredient’ was adopted in the UK complex intervention literature because early guidelines for the evaluation of complex interventions were based on the phases used to evaluate PIs [6]. The term ‘active ingredient’ refers to the components within an intervention that can be specifically linked to its effect on outcomes such that, if they were omitted, the intervention would be ineffective. For example, a cardiac rehabilitation intervention aimed at improving health-related behaviour associated with heart disease might focus on supporting smoking cessation, regular physical activity and a healthy diet [7]. Specific techniques used might include goal setting, providing information on consequences of behaviour and prompting self-monitoring of behaviour [8]. Provided these techniques have the potential to influence the health-related behaviour associated with heart disease (that is, causally influence outcomes) [7], they can be described as active ingredients. Therefore, throughout this article, the term ‘active ingredients’ is used to refer to these components in both PIs and NPIs. It is important to note that this is distinct from the mechanisms of action of interventions (the underlying reasons why the active ingredients have their particular effects) [7].

Accurate and comprehensive reporting of these components in titles and abstracts is essential [1,9]. Abstracts are more widely circulated than full-text articles [10] and are generally the most widely available parts of articles [11]. Consequently, their content can have a greater than anticipated impact [9]. Inadequate specification of key components in titles and abstracts can have serious implications for systematic reviews, which are considered the best sources of evidence about the effectiveness of interventions [12]. Since reviewers base their initial inclusion decisions on abstracts [9], inadequate specification may result in studies being inappropriately rejected from the review, thus compromising review validity. Additionally, many readers use abstract content to determine whether full-text retrieval is worthwhile [1,11,13], while in certain countries, many healthcare professionals have easy access to abstracts but not to full texts [1,9]. Therefore, it is important to investigate the quality of reporting in RCT abstracts.

The Consolidated Standards of Reporting Trials (CONSORT) guidelines [14] were developed to improve the quality of reporting of RCTs, and specific CONSORT guidelines (Table 1) have been developed for abstracts [1] and for NPIs [2], including surgical interventions, devices, rehabilitation packages and behaviour change interventions (BCIs) [15]. Owing to their complexity, NPIs are typically more difficult to standardize, describe and administer consistently than PIs while intervention success is often dependent on the expertise of the intervention providers [2,15].

It is plausible that PIs may be reported more precisely than NPIs in titles and abstracts, owing to the complexity of NPIs and the restrictive word limits of titles and abstracts. The active ingredients of a PI can usually be specified clearly in one phrase within a sentence (for example, ‘Zoledronic acid’ [16]). However, an NPI often cannot be specified so succinctly: as a result, intervention objectives are often specified, while active ingredients are often not reported (for example, ‘Self-management programme. The programme teaches patients medical, social and emotional self-management skills.’ [17]). Comparisons of full-text articles have found that PIs are more often described with enough detail to be faithfully reproduced [18], and that descriptions of the key components of NPIs are reported less often and less precisely than those of PIs [19]. However, we are not aware of any reviews that have systematically evaluated the reporting of NPIs as compared with PIs in titles and abstracts. 

Given the importance of accurate intervention reporting in titles and abstracts, an explicit comparison of reporting practices for PIs and NPIs would be useful for a number of reasons. Firstly, to establish whether there are similar disparities in the quality of abstract reporting between PIs and NPIs as there are for full-text articles. Reviews of full-text articles have illustrated how reporting of NPIs can be improved based on PI reporting practices; a comparative review of abstracts could similarly illustrate how the reporting of abstracts describing NPIs could be improved.

Title and abstract reporting practices for BCIs, a subset of NPIs that aim to modify health-related behaviour [20,21], require special scrutiny. The CONSORT reporting guidelines have been adopted by most behavioural research journals [22]. Although Davidson and colleagues [3] proposed additional guidelines for reporting BCIs, these do not include specific recommendations for abstracts. It has been argued that full-text reports of BCIs usually do not fully comply with these guidelines [22]. However, to our knowledge, title and abstract reporting practices for BCIs have not been reviewed systematically.

These issues highlight that an explicit comparison of title and abstract reporting practices of PIs, NPIs and BCIs is necessary to clarify how reporting of NPIs and BCIs can be improved. This illustrative review compares the specification of interventions in titles and abstracts of published reports of RCTs. It was hypothesized that active ingredients would be reported more often for PIs than NPIs, and for other NPIs than BCIs.

## Methods

### Data sources and search methods

Studies were sampled from the *British Medical Journal*, *The Journal of the American Medical Association*, *The New England Journal of Medicine*, *The Lancet* and *Annals of Behavioral Medicine* (*ABM*). The first four journals each have a high impact factor [23], which is associated with high methodological quality of articles [24]. These journals consequently provide a source of high-quality reports. *ABM* is the most influential behavioural research journal (with an impact factor in 2010 of 3.984) and is assumed to exemplify high-quality reporting of BCIs. This journal was selected so that an adequate sample of high-quality BCI reports could be included. This choice of a limited range of journals is in accordance with previous comparisons of reporting practices where the goal is not to be exhaustive but to highlight the issues inherent in the reporting of NPIs [18].

A search strategy (Additional file 1) comprising both Medical Subject Headings and text words was designed and executed in MEDLINE (1946 to March 2011 Week 3) and Embase (1980 to 2011 Week 11), using the Ovid interface. The search strategy identified RCTs and specific journal titles, imposed a date restriction to reflect recent reporting practice (January 2009 to March 2011) and used the controlled vocabulary term ‘drug therapy’ to distinguish between PI and NPI reports.

### Inclusion criteria

Only RCTs or randomized studies were considered for inclusion. Eligible interventions were PIs or NPIs. Eligible comparator interventions were control treatments or other PIs or NPIs. There were no restrictions on the types of participant, outcome measure or length of follow-up. Papers that were not the primary report of a study (for example secondary analyses of trial data) were excluded. CONSORT recommends that although space limitations restrict abstract content, interventions should be described with enough detail to be fully understood [1]. This should be the objective of primary research reports. Intervention description is not necessarily the main focus of secondary research reports, and so a full description of the intervention may legitimately be omitted from the title or abstract.

### Study selection and data extraction

One researcher (NM) conducted the search and randomly selected 210 papers (10 for piloting the method and 200 for the main analysis) [25]. Since this review explored differences in reporting practices, the inclusion of 210 papers was judged to be representative of reporting variations in high-quality journals. Additionally, this number of papers would be unlikely to violate the statistical assumptions of the proposed chi-square analyses. To ensure that the sample was representative of the population of studies considered, papers were sampled proportionally to reflect the proportions of PI and NPI reports typically published by these journals, rather than sampling an equal number of papers from each journal. The calculations performed to determine the numbers of PI and NPI reports to be sampled from each journal are included in Additional file 2. One researcher (NM) screened all titles and abstracts to determine eligibility. Any uncertainties were resolved through discussion with the research team.

A data extraction form (Additional file 3) was developed by one researcher (NM), in collaboration with the research team, using the *Cochrane Handbook for Systematic Reviews of Interventions*[26]. The form contained a classification scheme, used to extract details of intervention components, created using CONSORT guidelines. The form was piloted by one researcher (NM) and a colleague (a trainee health psychologist). Data extracted in relation to components reported were converted into frequency data, and inter-coder agreement was assessed for each of the ten pilot papers using the Kappa statistic. Kappa values ranged between 0.44 and 0.76, indicating moderate or substantial agreement for all ten papers [27]. Minor changes were made to the form following piloting. Piloting indicated that the classification scheme could be used to extract relevant data from titles and abstracts and that the scheme was fully comprehensive in terms of the components of interventions typically reported in titles and abstracts. Data extraction was carried out by one researcher (NM) and any uncertainties were resolved through discussion with the research team.

### Data analysis

The studies were coded for the presence or absence of study components, resulting in frequency data that were analyzed using SPSS version 17.0. We recognized that coding the presence or absence of active ingredients of NPIs might be a matter of personal judgement, and so two authors (JJF and NM), independently coded the presence or absence of active ingredients for all NPI titles. JJF was blinded to the original coding, while NM re-coded these titles without reviewing the original coding. Discrepancies were agreed by consensus. This ensured consistency between our definition of active ingredients and the resultant coding.

Chi-square tests were performed for titles and abstracts separately to investigate the associations between intervention type (PI/NPI) and reporting of the intervention’s active ingredients (yes/no). This was repeated to compare BCIs with other NPIs, which was a pre-specified subgroup analysis. Exploratory chi-square analyses illustrating further differences in reporting practices were also conducted.

## Results

### Study selection

**Figure 1:**
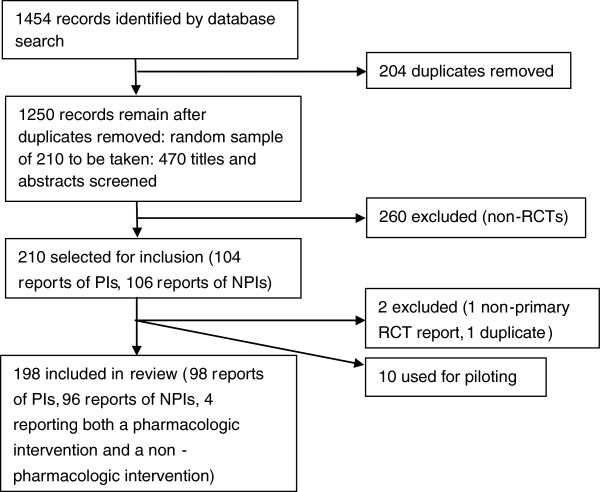
**Flow chart of identification and selection of included studies.** NPI = non-pharmacologic intervention; PI = pharmacologic intervention; RCT = randomised controlled trial.

The search identified 1,250 papers after deduplication, from which the random sample of 210 was taken. After removal of the ten papers used for piloting, one duplicate and one non-primary RCT report, 198 papers were reviewed, of which 98 were PI reports and 96 were NPI reports. Four papers reported both intervention types and were not included in the analyses. Details of the search are summarized in Figure 1.

### Characteristics of included studies

Of the 98 PI reports, 87 evaluated a drug, 10 evaluated a vaccine and one evaluated both a drug and a vaccine. The interventions described within the 96 NPI reports were categorized as either behaviour change (when aimed at changing behaviour; 41 reports); surgery (10 reports); device (9 reports); screening (5 reports); food supplementation (involving a preparation aimed at supplementing diet; 5 reports); or rehabilitation (when aimed at restoring health and/or functioning; 5 reports). These categories were created based on the studies used in the piloting phase.

An intervention type could not be specified for five papers (these were excluded from all subgroup analyses as it was unclear whether or not they were BCIs). The remaining 16 papers reported evaluations of interventions that could not be classified into any of the six categories mentioned previously (see Additional file 4). Five papers that reported more than one trial were excluded from all analyses as a full intervention description might not have been provided. The basic characteristics of included studies are presented in Additional file 4.

### Analysis of included studies

**Figure 2:**
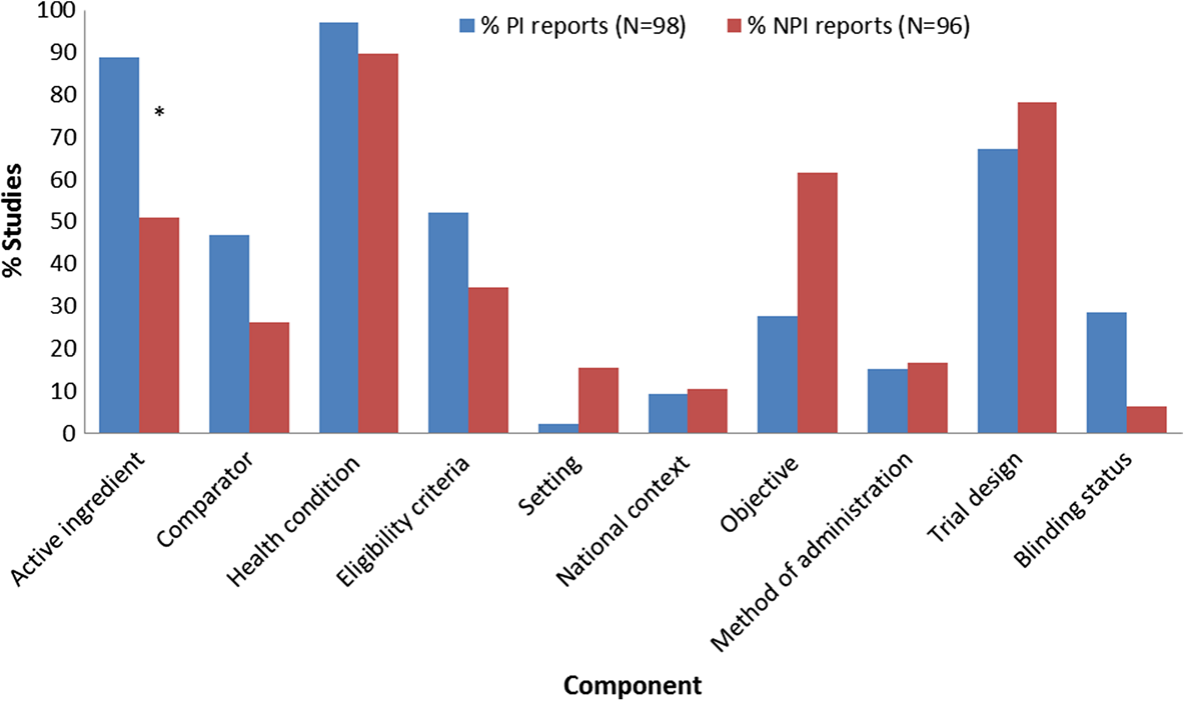
**Percentage of titles reporting specific study components.** NPI = non-pharmacologic intervention; PI = pharmacologic intervention. * Statistical significance of differences in percentages of studies were tested for the ‘active ingredients’ component only: P< 0.001. Note: All other study components reported by <10% of either the PI or NPI reports so are not included in figure (primary outcome; dose/intensity; frequency of treatment; duration of treatment; comparator dose/ intensity; comparator method of administration; comparator frequency of treatment; comparator duration of treatment; timing of outcome assessment; trial phase; trial registration; intervention providers).

**Figure 3:**
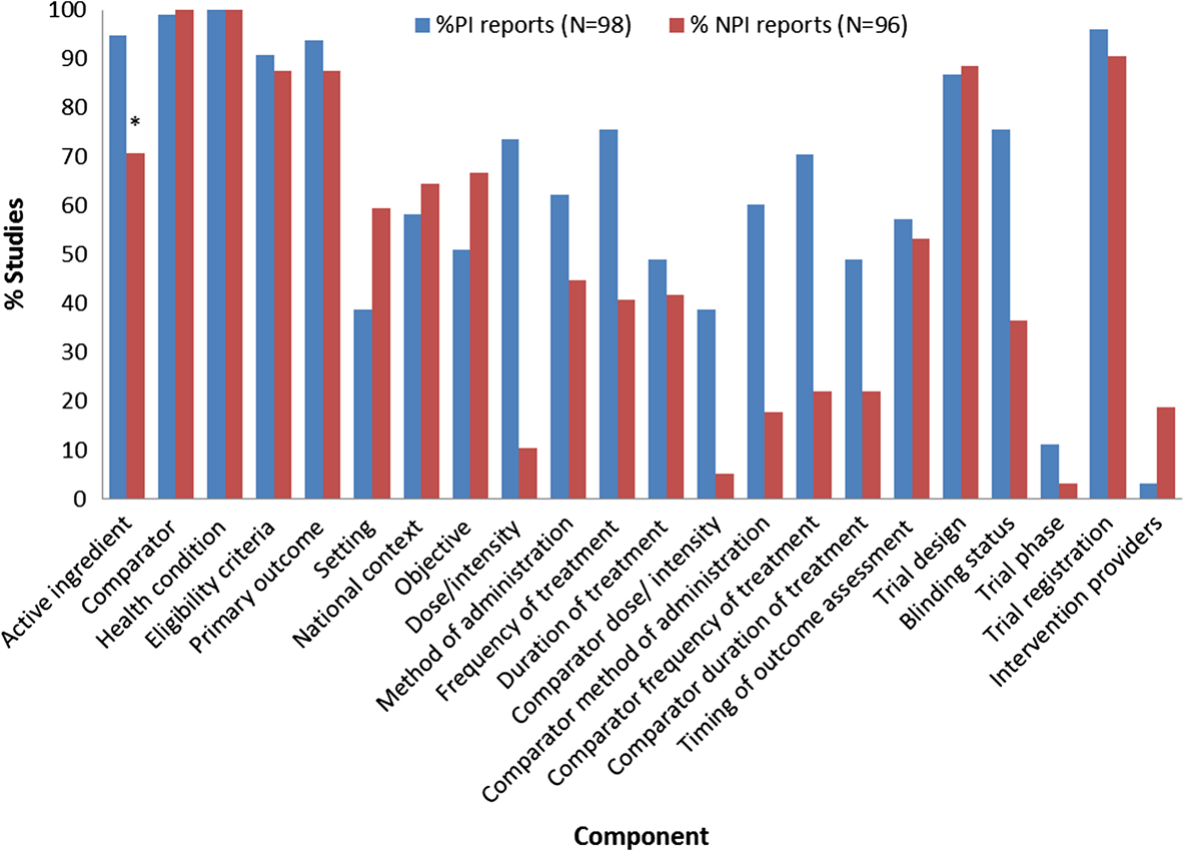
**Percentage of abstracts reporting specific study components.** NPI = non-pharmacologic intervention; PI = pharmacologic intervention. * Statistical significance of differences in percentages of studies were tested for the ‘active ingredients’ component only: P< 0.001.

Table 2 illustrates intervention component coding. Figures 2 and 3 show the percentages of PI and NPI titles and abstracts reporting specific intervention components. In titles, PI articles more often reported the active ingredients, comparator interventions, eligibility criteria and blinding status; NPI articles more often reported the setting and intervention objectives (Figure 2). In abstracts, PI articles more often reported the active ingredients, dose or intensity, method of administration, frequency of treatment, duration of treatment and blinding status; NPI articles more often included the settings, intervention objectives and intervention providers (Figure 3).

**Table 2:** Example of study component coding

	**Pharmacologic intervention title: ‘Vitamins C and E for prevention of pre-eclampsia in women with type 1 diabetes (DAPIT): a randomized placebo-controlled trial’ **[28]	**Non-pharmacologic intervention title: ‘Treatment of childhood obesity by retraining eating behaviour: randomized controlled trial’ **[29]
Active ingredients	Vitamins C and E	Not reported
Comparator	Placebo	Not reported
Health condition	Pre-eclampsia	Obesity
Eligibility criteria	Women with type 1 diabetes	Children
Objective	Prevention of pre-eclampsia	Retraining eating behaviour
Trial design	Randomized controlled trial	Randomized controlled trial

Active ingredients were named in the majority of titles (82/93, 88.17%) and abstracts (88/93, 94.62%) of PI reports, but less frequently in the titles (49/96, 51.04%) and abstracts (68/96, 70.83%) of NPI reports. There was a significant association between intervention type and reporting of the active ingredients for titles (*χ*^2^(1) = 28.90; *P* < 0.001) and abstracts (*χ*^2^(1) = 16.94; *P* < 0.001).

BCI reports named active ingredients in just over a third of titles (15/41, 36.59%) and just over half of abstracts (23/41, 56.10%). Reports of other NPIs named active ingredients in two-thirds of the titles (33/50, 66.00%) and in the majority of abstracts (43/50, 86.00%). There was a significant association between NPI intervention type and reporting of the active ingredients for titles (*χ*^2^(1) = 6.68; *P* = 0.010) and for abstracts (*χ*^2^(1) = 8.66; *P* = 0.003). We discovered that while *ABM* limits abstracts to 150 words [30], the abstract limits of the other included journals range between 250 and 300 words [31-34]. Consequently, we conducted a sensitivity analysis by removing the six papers published in *ABM* but we still found a significant association between NPI intervention type and reporting of the active ingredients for titles (*χ*^2^(1) = 5.79; *P* = 0.016), and abstracts (*χ*^2^(1) = 10.50; *P* = 0.001).

We conducted exploratory analyses to investigate whether intervention objectives or settings might be reported in place of active ingredients, as is sometimes the case for NPIs (for example, describing an intervention as ‘a weight-loss intervention’ rather than as a ‘cognitive behavioural therapy intervention to support weight loss’). The majority of PI articles reporting an objective in the title also reported the active ingredients (16/26, 61.54%) and similarly for abstracts (44/48, 91.67%). However, approximately a third of NPI articles reporting an objective in the title also reported the active ingredients (19/59, 32.20%) while nearly two-thirds did so in abstracts (40/64, 62.50%). There was a significant association between intervention type and reporting of active ingredients with objectives for titles (*χ*^2^(1) = 5.26; *P* = 0.022) and abstracts (*χ*^2^(1) = 10.94; *P* = 0.001).

The trial setting was reported in the titles of two PI reports and 15 NPI reports, 11 of which did not report active ingredients. The vast majority of PI articles reporting the setting in the abstract also reported the active ingredients (36/38, 94.74%), whereas nearly two-thirds of the NPI articles reporting the setting in the abstract did so (36/57, 63.16%). There was a significant association between intervention type and reporting of active ingredients with settings for abstracts (*χ*^2^(1) = 10.73; *P*= 0.001). In summary, it appears that when intervention objectives and settings are reported for NPIs, active ingredients are often missing.

In the abstracts, details of the intervention providers were reported for three PIs and 18 NPIs. The three PI articles reported the involvement of healthcare professionals. Fourteen of the 18 NPI abstracts reported who provided the intervention, while four (which were all reports of BCIs) provided additional information on the training delivered.

## Discussion

Active ingredients were reported significantly less often in titles and abstracts for NPIs than PIs, and for BCIs than other types of NPIs. These results are in accordance with those described previously from reviews comparing full-text PI and NPI reports [18,19]. This indicates that improvements are required in the reporting of NPIs in titles and abstracts, and that, in accord with much of the literature [35], titles and abstracts of BCI reports in particular lack a detailed intervention description. Without this key information, readers are left with an incomplete picture of what the intervention involved [14]. On the basis of our findings, we recommend that all authors routinely report active ingredients in titles and abstracts, and that all journal editors routinely require this.

It is possible that variation in reporting is attributable to the differences in the existing nomenclature for these interventions. Agreed labels (drug names) are used for the same PI [18,36], making specification more straightforward. NPIs, however, usually involve procedures that can be labelled and performed in a variety of ways [2,19], as illustrated in Table 3. Here, the same term is used to label the interventions in the title, but very different descriptions are provided in the articles. A set of agreed labels and definitions of active ingredients of complex interventions, which is currently lacking [36], would help to standardize reporting. Indeed, a system for specifying the exact techniques used in BCIs has recently been developed [21].

**Table 3:** **Different descriptions of ‘behavioural counselling’ intervention in two studies (adapted from [**[37]**])**

**Study 1 **[38]	**Study 2 **[39]
Feedback (on diaries)	Assessment of readiness to change
Reinforcement	Attitude change
Recommendations for change	Goal setting
Answers to questions	Specific behavioural advice
General support	

Furthermore, abstract space limitations might contribute to these reporting differences. The CONSORT guidelines for abstracts state that 250 to 300 words are sufficient to report all recommended items [1]. However, it is likely that specifying PIs requires fewer words than NPIs. Another study of abstract reporting quality found that a greater abstract word count was associated with higher reporting quality for structured abstracts [13]. The 150-word limit for abstracts imposed by *ABM* therefore seems unsuitable. Journals could consider increasing abstract word limits to facilitate higher-quality abstract reporting, particularly for NPIs.

The exploratory analyses suggested that when intervention objectives and settings are reported for NPIs, active ingredients are often missing. This concurs particularly with previous behavioural research, where greater focus is often placed on reporting such aspects as the intervention objectives, trial setting, method of administration and intervention providers [3,8]. Although these are important, describing interventions in this way can make different intervention content indistinguishable and thus make it difficult to determine the specific techniques that might be critical to intervention effectiveness [8].

Only 59% of the NPI reports mentioned the setting in the abstract. This seems low, given that the settings in which NPIs are administered are likely to interact with the intervention’s active ingredients to influence its effectiveness [40]. In addition, the effectiveness of an NPI can be influenced by the skills, experience and enthusiasm of the providers [18]. The CONSORT guidance for NPIs states that the number of providers involved in intervention delivery and details of their expertise should be reported in the abstract [2]. However, only 19% of the NPI reports contained any information about intervention providers in the abstract. Clearly, reporting of these components also requires improvement.

This is the first review to compare reporting of specific components of PIs and NPIs in titles and abstracts, and is therefore the first to show that active ingredients are reported more often in titles and abstracts for PIs than NPIs. There are, however, important limitations of this work. It was assumed that all included studies were of a high quality because they were published in high-impact journals: this cannot be confirmed, since a full quality assessment was not conducted. This was beyond the scope of this project. Our findings may be generalizable to other general medical journals of similar quality; however, the generalizability of our findings to other types of journal or conference abstracts may be limited. Journal recommendations for abstract word length were not verified at the outset of the study, and so there was imbalance between the journals targeted. Abstract limits were not checked because, as specified previously, our criterion for journal selection was high journal quality.

Data extraction and analysis were carried out by one reviewer. However, coding the active ingredients of NPIs was conducted by two reviewers, and there was general consensus regarding the data extracted during piloting. Study dropout must also be highlighted; one duplicate, one non-primary RCT report and four reports describing both intervention types were removed from the analysis. This resulted in unbalanced numbers of papers in the PI and NPI categories; these excluded papers could have been replaced, such that an equal number of papers in each group were analyzed.

The many differences between PIs and NPIs might make comparison difficult to interpret; however, existing abstract reporting guidelines were designed to apply to both intervention types, implying that both abstract types should be written to a similar standard. We have shown that this is not the case in these journals. Owing to the proportional sampling strategy used, only six articles published in *ABM* were included. The sample reflected the relative frequency of publication: fewer RCTs are published in *ABM* than in the other journals; therefore, fewer were retrieved and sampled. Although this reflects the relative frequency of publication, this is a limitation, given that this journal was specifically selected for inclusion of high-quality reports of BCIs. 

One final important limitation concerns the link between abstract and full-text reporting. Reviews of full-text articles [18,19] and reviews of abstracts [9,10,13,41] have highlighted deficiencies in reporting. However, more research is required to verify whether the quality of abstract reporting is linked to that of full-text reporting. It is therefore not clear whether findings similar to ours would be obtained if full-text articles were reviewed in the same way: this is an important follow-up evaluation.

This review has several strengths. No intervention types were excluded, so the results apply broadly to reporting of PIs and NPIs. Included studies were recently published in high-impact peer-reviewed journals, so it appears that there are problems with very recent reporting practice even for high-quality reports. Finally, this review is unique in that it comprises an explicit comparison of the reporting of specific intervention components in titles and abstracts. The study of abstract quality is a fairly recent development [9], with most reviews focused on reporting of trial components [9,10,13]. Many studies use the CONSORT abstract reporting guidelines to evaluate quality. CONSORT specifies that intervention details should be reported, but gives no guidance on *how* they should be reported: this is the first review of abstracts to focus specifically on the level of detail provided regarding different types of intervention.

## Conclusions

Active ingredients were more frequently reported in titles and abstracts for PIs than for NPIs, and more frequently for other NPIs than for BCIs. This review identifies the need for improved reporting of NPIs, and BCIs in particular. The creation of agreed labels and definitions for active ingredients of complex interventions, particularly BCIs, would contribute towards clearer reporting.

## Abbreviations

ABM: Annals of Behavioral Medicine; BCI: Behaviour change intervention; CONSORT: Consolidated Standards of Reporting Trials; NPI: Non-pharmacologic intervention; PI: Pharmacologic intervention; RCT: Randomized controlled trial.

## Competing interests

The authors declare that they have no competing interests.

## Authors’ contributions

JJF conceived the study and participated in its design and coordination. NM participated in the design of the study, helped design and execute the search strategy, conducted the title and abstract screening, extracted all data and performed the statistical analysis. EMD participated in the study design and coordination. FS helped design and execute the search strategy and provided advice on reference management. All authors helped to draft the manuscript, and read and approved the final version of the manuscript.

## Supplementary Material

Additional file 1 Search strategy. Click here for file

Additional file 2 Sampling calculations. Click here for file

Additional file 3 Data extraction form. Click here for file

Additional file 4 Characteristics of included studies. Click here for file
