# Supplementary material for: Active ingredients are reported more often for pharmacologic than non-pharmacologic interventions: an illustrative review of reporting practices in titles and abstracts
Source: Trials. 2013 May 20;14:146. doi: 10.1186/1745-6215-14-146 (PMC3663666; doi:10.1186/1745-6215-14-146)
Supplement: Additional file 1 — Search strategy. [file 1745-6215-14-146-S1.doc]

**Search Strategy**

MEDLINE (1948 to March Week 3 2011)

Embase (1980 to 2011 Week 11)

Ovid multifile search: http://shibboleth.ovid.com/

1. exp Clinical Trials as Topic/ use mesz
2. clinical trial/ use emez
3. Randomized Controlled Trial/
4. randomized controlled trial.pt.
5. randomization/ use emez
6. random$.ti,ab.
7. control group/ use emez
8. (control adj3 group$).ti,ab.
9. attention control.ti,ab.
10. Placebos/ use mesz
11. placebo/ use emez
12. placebo$.ti,ab.
13. or/1-12
14. british medical journal.jn.
15. bmj.jn.
16. journal of the american medical association.jn.
17. jama.jn.
18. new england journal of medicine.jn.
19. nejm.jn.
20. lancet.jn.
21. annals of behavio?ral medicine.jn.
22. annals of behavio?ral medicine a publication of the society of behavio?ral medicine.jn.
23. ann behav med.jn.
24. or/14-23
25. 13 and 24
26. limit 25 to yr="2009-current"
27. (letter or review or comment or conference$ or editorial).pt.
28. 26 not 27
29. (animals/ or nonhuman/) not humans/
30. 28 not 29
31. remove duplicates from 30
32. dt.fs.
33. (14 or 15) and 31 and 32
34. (16 or 17) and 31 and 32
35. (18 or 19) and 31 and 32
36. 20 and 31 and 32
37. ((14 or 15) and 31) not 32
38. ((16 or 17) and 31) not 32
39. ((18 or 19) and 31) not 32
40. (20 and 31) not 32
41. (or/21-23) and 31

|  |  |  |
| --- | --- | --- |
|  |  |  |
|  |  |  |
|  |  |  |
|  |  |  |
|  |  |  |
|  |  |  |
|  |  |  |
|  |  |  |
|  |  |  |
