# Supplementary material for: Active ingredients are reported more often for pharmacologic than non-pharmacologic interventions: an illustrative review of reporting practices in titles and abstracts
Source: Trials. 2013 May 20;14:146. doi: 10.1186/1745-6215-14-146 (PMC3663666; doi:10.1186/1745-6215-14-146)
Supplement: Additional file 2 — Sampling calculations. [file 1745-6215-14-146-S2.doc]

**Sampling Calculations**

Proportional sampling calculation: Number sampled= (number of papers retrieved by search strategy for the specific journal-intervention type combination/total papers retrieved by search strategy) x 210*

| **Journal (intervention type)** | **Calculation** | **Number of papers to be sampled** |
| --- | --- | --- |
| BMJ (PI) | (126/1250) x 210 = 21.168 | 21 |
| JAMA (PI) | (57/ 1250) x 210 = 9.576 | 10 |
| NEJM (PI) | (261/1250) x 210 = 43.848 | 44 |
| Lancet (PI) | (125/1250) x 210 = 21 | 21 |
| BMJ (NPI) | (234/ 1250) x 210 = 39.312 | 39 |
| JAMA (NPI) | (171/1250) x 210 = 28.728 | 29 |
| NEJM (NPI) | (97/ 1250) x 210 = 16.296 | 16 |
| Lancet (NPI) | (142/1250) x 210 = 23.856 | 24 |
| ABM | (37/1250) x 210 = 6.216 | 6 |

ABM = *Annals of Behavioral Medicine*; BMJ = *British Medical Journal*; JAMA = *Journal of the American Medical Association*; NEJM = *New England Journal of Medicine*; NPI = Non-pharmacologic intervention; PI = Pharmacologic intervention

* 210 = target number of papers for inclusion in review
