# Supplementary material for: Active ingredients are reported more often for pharmacologic than non-pharmacologic interventions: an illustrative review of reporting practices in titles and abstracts
Source: Trials. 2013 May 20;14:146. doi: 10.1186/1745-6215-14-146 (PMC3663666; doi:10.1186/1745-6215-14-146)
Supplement: Additional file 3 — Data extraction form. [file 1745-6215-14-146-S3.doc]

**Data Extraction Form**

Data extraction sheet version number: 3 (08 May 2011)

Reviewer ID:

Date:

Study ID:

Author:

Study title:

Journal:

Year/volume/issue/page no’s:

Type of Intervention:

Pharmacologic: Drug

Vaccine

Other: Please specify

Nonpharmacologic: Surgical

Device

Behaviour change

Screening

Other: Please specify:

Verification of study eligibility:

RCT/randomised study

Further comment if applicable:

Total number of participants:

Total number of comparison groups:

Total number of study sites:

Total number of words in title:

Total number of words in abstract:

**Information in the title:**

| **Keyword** | **Information** | **Line Ref.** |
| --- | --- | --- |
| Active ingredient |  |  |
| Comparator | 1.  2.  3. |  |
| Health condition |  |  |
| Eligibility criteria |  |  |
| Primary outcome |  |  |
| Setting |  |  |
| National context |  |  |
| Objective |  |  |
| Dose/intensity |  |  |
| Method of administration |  |  |
| Frequency of treatment |  |  |
| Duration of treatment |  |  |
| Comparator dose/ intensity |  |  |
| Comparator method of administration |  |  |
| Comparator frequency of treatment |  |  |
| Comparator duration of treatment |  |  |
| Timing of outcome assessment |  |  |
| Trial design |  |  |
| Blinding status  (circle all that apply) | Participants / intervention providers / outcome assessors / no blinding / unclear |  |
| Trial phase |  |  |
| Trial registration |  |  |
| Intervention providers |  |  |

**Information in the abstract:**

| **Keyword** | **Information** | **Line Ref.** |
| --- | --- | --- |
| Active ingredient |  |  |
| Comparator | 1.  2.  3. |  |
| Health condition |  |  |
| Eligibility criteria |  |  |
| Primary outcome |  |  |
| Setting |  |  |
| National context |  |  |
| Objective |  |  |
| Dose/intensity |  |  |
| Method of administration |  |  |
| Frequency of treatment |  |  |
| Duration of treatment |  |  |
| Comparator dose/ intensity |  |  |
| Comparator method of administration |  |  |
| Comparator frequency of treatment |  |  |
| Comparator duration of treatment |  |  |
| Timing of outcome assessment |  |  |
| Trial design |  |  |
| Blinding status  (circle all that apply) | Participants / intervention providers / outcome assessors / no blinding / unclear |  |
| Trial phase |  |  |
| Trial registration |  |  |
| Intervention providers |  |  |

Notes:
