# Supplementary material for: Active ingredients are reported more often for pharmacologic than non-pharmacologic interventions: an illustrative review of reporting practices in titles and abstracts
Source: Trials. 2013 May 20;14:146. doi: 10.1186/1745-6215-14-146 (PMC3663666; doi:10.1186/1745-6215-14-146)
Supplement: Additional file 4 — Characteristics of included studies. [file 1745-6215-14-146-S4.doc]

**Characteristics of Included Studies**

Studies evaluating pharmacologic interventions

| **First author, journal, year, volume, first page** | **Patient group and intervention target** | **Sample size** | **Intervention type** | **Active ingredients** |
| --- | --- | --- | --- | --- |
| Achnan, BMJ, 2009, 339, 283 | Malaria in children aged 6-59 months | 175 | Drug | Quinine |
| Cohen, BMJ, 2009, 338, 986 | Patients with greater trochanteric pain syndrome | 65 | Drug | Corticosteroid & local anaesthetic |
| Ermers, BMJ, 2009, 338, b897 | Wheeze in infants less than 13 months with respiratory syncytial virus infections | 243 | Drug | Corticosteroid; extra fine hydrofluoroalkane beclometasone dipropionate |
| Heal, BMJ, 2009, 338, 211 | Wound infection prevention in patients having minor dermatological surgery | 972 | Drug | Chloramphenicol |
| Hijazi, BMJ, 2009, 338, 457 | Pain prevention in adult patients requiring intravenous cannulation | 201 | Drug | Alkane; blend of propane, butane and pentane |
| Aaby, BMJ, 2010, 341, 1262 | Mortality prevention in children aged 4.5 months receiving measles vaccination | 6648 | Vaccine | Edmonston-Zagreb vaccine |
| Austin, BMJ, 2010, 341, 927 | Mortality prevention in patients with chronic obstructive pulmonary disease | 405 | Drug | Oxygen |
| Benn, BMJ, 2010, 340, 636 | Mortality prevention in low birthweight neonates | 1717 | Drug/vaccine | Vitamin A & BCG vaccine |
| Blomberg, BMJ, 2010, 341, 81 | Wound infection prevention in patients undergoing percutaneous endoscopic gastrostomy | 234 | Drug | Sulfamethoxazole & trimethoprim (also known as co-trimoxazole/ bactrim) |
| Chen, BMJ, 2010, 341, 4024 | Patients with remitted first episode psychosis | 178 | Drug | Quetiapine |
| de Jager, BMJ, 2010, 340, 2181 | Vitamin B-12 deficiency in patients with type 2 diabetes | 390 | Drug | Metformin |
| Fagerstrom, BMJ, 2010, 341, 1259 | Smokeless tobacco use in adults aged >=18 | 431 | Drug | Varenicline |
| Maruyama, BMJ, 2010, 340, 579 | Pneumococcal pneumonia prevention in nursing home residents | 1006 | Vaccine | 23-valent polysaccharide vaccine |
| Roth, BMJ, 2010, 340, 749 | Mortality prevention in children aged 19 months receiving BCG revaccination | 2871 | Vaccine | BCG revaccination |
| Sterry, BMJ, 2010, 340, 147 | Patients with psoriasis and psoriatic arthritis | 752 | Drug | Etanercept |
| **First author, journal, year, volume, first page** | **Patient group and intervention target** | **Sample size** | **Intervention type** | **Active ingredients** |
| Tufail, BMJ, 2010, 340, 1398 | Patients with neovascular age related macular degeneration | 131 | Drug | Bevacizumab |
| Waddington, BMJ, 2010, 340, 1292 | Influenza prevention in children aged 6 months to 12 years | 943 | Vaccine | AS03B (tocopherol based oil in water emulsion) adjuvanted split virion vaccine |
| Bawaskar, BMJ, 2011, 342, 153 | Patients older than six months with scorpion envenomation | 70 | Drug | Not provided |
| Gaziano, JAMA, 2009, 301, 52 | Prostate and total cancer prevention in male physicians aged >=50 | 14641 | Drug | Vitamins E & C |
| Manzoni, JAMA, 2009, 302, 1421 | Late-onset sepsis prevention in very low birthweight infants | 472 | Drug | bovine lactoferrin & lactobacillus rhamnosus GG |
| Meuwese, JAMA, 2009, 301, 1131 | Carotid atherosclerosis in patients with familial hypercholesterolemia | 892 | Drug | Pactimibe (plus standard lipid-lowering therapy) |
| Meyhoff, JAMA, 2009, 302, 1543 | Surgical site infection & pulmonary complication prevention in patients undergoing abdominal surgery | 1400 | Drug | Oxygen |
| Porco, JAMA, 2009, 302, 962 | Mortality prevention in patients treated for trachoma | 66404 | Drug | Azithromycin |
| Schnuelle, JAMA, 2009, 302, 1067 | Graft function in patients receiving a kidney transplant | 487 | Drug | Dopamine |
| Smith, JAMA, 2009, 302, 1444 | Altitude-induced hypoxic pulmonary hypertension in: sea-level resident men aged 19-60 (study 1): high-altitude resident men aged 30-59 (study 2) | 22 and 11 | Drug | Study 1: Fe(III)-hydroxide sucrose; Study 2: staged isovolemic venesection of 2 L of blood, Fe(III)-hydroxide sucrose, placebo |
| van Gils, JAMA, 2009, 302, 159 | Nasopharyngeal pneumococcal carriage in healthy newborns | 1003 | Vaccine | 7-valent pneumococcal conjugate vaccine |
| Annane, JAMA, 2010, 303, 341 | Septic shock in adults with multiple organ dysfunction | 509 | Drug | Insulin & hydrocortisone |
| Foell, JAMA, 2010, 303, 1266 | Patients with juvenile idiopathic arthritis in remission | 364 | Drug | Methotrexate |
| Hiemstra, JAMA, 2010, 304, 2381 | Patients with newly-diagnosed antineutrophil cytoplasmic antibody-associated vasculitis aged 18 to 75 at diagnosis | 156 | Drug | Mycophenolate mofetil |

| **First author, journal, year, volume, first page** | **Patient group and intervention target** | **Sample size** | **Intervention type** | **Active ingredients** |
| --- | --- | --- | --- | --- |
| House, JAMA, 2010, 303, 1603 | Diabetic nephropathy in patients with type 1 or 2 diabetes | 238 | Drug | Folic acid, vitamin B(6), vitamin B(12) |
| Makrides, JAMA, 2010, 304, 1675 | Maternal depression & neurodevelopment of children in women less than 21 weeks’ gestation | 2399 | Drug | Docosahexaenoic acid-rich fish oil |
| Steg, JAMA, 2010, 304, 1339 | Bleeding & major vascular access-site complication prevention in patients undergoing percutaneous coronary intervention | 2026 | Drug | Unfractionated heparin |
| Wilkens, JAMA, 2010, 304, 45 | Pain-related disability prevention in patients with chronic low back pain & degenerative lumbar osteoarthritis older than 25 | 250 | Drug | Glucosamine |
| Gladwin, JAMA, 2011, 305, 893 | Patients with sickle cell pain crisis | 150 | Drug | Nitric oxide |
| Astrup, Lancet, 2009, 374, 1606 | Obese individuals without type 2 diabetes aged 18-65 | 564 | Drug | Liraglutide |
| Becker, Lancet, 2009, 373, 919 | Bleeding prevention in patients undergoing percutaneous coronary intervention aged 45 or older | 1030 | Drug | SCH 530348 |
| Carr, Lancet, 2009, 373, 226 | Systemic sepsis prevention in preterm neonates below the 10th centile for birthweight | 280 | Drug | Granulocyte-macrophage colony stimulating factor |
| Comi, Lancet, 2009, 374, 1503 | Progression to clinically definite multiple sclerosis in patients with a clinically isolated syndrome | 481 | Drug | Glatiramer acetate |
| Ferguson, Lancet, 2009, 373, 1264 | Skin scar improvement in healthy adults | Unclear | Drug | Avotermin |
| Gosling, Lancet, 2009, 374, 1521 | Malaria prevention in infants aged 8-16 weeks | 2419 | Drug | Sulfadoxine & pyrimethamine |
| Konstam, Lancet, 2009, 374, 1840 | Patients with heart failure | 3846 | Drug | Losartan |
| Munoz, Lancet, 2009, 373, 1949 | Human papillomavirus prevention in women aged 24-45 | 3819 | Vaccine | Quadrivalent human papillomavirus (types 6, 11, 16, 18) L1 virus-like-particle vaccine |

| **First author, journal, year, volume, first page** | **Patient group and intervention target** | **Sample size** | **Intervention type** | **Active ingredients** |
| --- | --- | --- | --- | --- |
| Pirker, Lancet, 2009, 373, 1525 | Advanced non-small-cell lung cancer in chemotherapy-naive patients aged >=18 | 1125 | Drug | Cisplatin, vinorelbine, cetuximab |
| Priotto, Lancet, 2009, 374, 56 | Human African trypanosomiasis in patients aged >=15 | 287 | Drug | Nifurtimox-eflornithine combination therapy |
| Teerlink, Lancet, 2009, 373, 1429 | Patients with acute heart failure | 234 | Drug | Relaxin (& standard care) |
| Weber, Lancet, 2009, 374, 1423 | Patients with treatment-resistant hypertension | 379 | Drug | Darusentan |
| Yusuf, Lancet, 2009, 373, 1341 | Risk factors in individuals without cardiovascular disease aged 45-80 | 2053 | Drug | Polycap (polypill): thiazide, atenolol, ramipril, simvastatin, aspirin |
| Armah, Lancet, 2010, 376, 606 | Severe rotavirus gastroenteritis prevention in infants aged 4-12 weeks | 5468 | Vaccine | Pentavalent vaccine |
| Armitage, Lancet, 2010, 376, 1658 | LDL cholesterol lowering in adults with a history of myocardial infarction aged 18-80 | 12064 | Drug | Simvastatin |
| Bouadma, Lancet, 2010, 375, 463 | Antibiotic exposure reduction in patients in intensive care units aged >=18 | 630 | Drug | Not provided |
| de Bono, Lancet, 2010, 376, 1147 | Men with metastatic castration-resistant prostate cancer | 755 | Drug | Prednisone & cabazitaxel |
| Eron, Lancet, 2010, 375, 396 | HIV-infected patients aged >=18 | 707 | Drug | Raltegravir (& background therapy: at least two nucleoside/nucleotide reverse transcriptase inhibitors) |
| Glasier, Lancet, 2010, 375, 555 | Unintended pregnancy prevention in women requesting emergency contraception | 2221 | Drug | Ulipristal acetate |
| Hovorka, Lancet, 2010, 375, 743 | Patients with type 1 diabetes aged 5-18 | 19 | Drug | Insulin |
| Kirkwood, Lancet, 2010, 375, 1640 | Mortality prevention in women aged 15-45 | 207781 | Drug | Vitamin A |
| Lassen, Lancet, 2010, 375, 807 | Venous thromboembolism prevention after knee replacement | 3057 | Drug | Apixaban |

| **First author, journal, year, volume, first page** | **Patient group and intervention target** | **Sample size** | **Intervention type** | **Active ingredients** |
| --- | --- | --- | --- | --- |
| McCance, Lancet, 2010, 376, 259 | Pre-eclampsia prevention in women with type 1 diabetes aged >=16 | 762 | Drug | Vitamins C & E (alpha-tocopherol) |
| Mercier, Lancet, 2010, 376, 346 | Bronchopulmonary dysplasia prevention in preterm infants requiring surfactant or continuous positive airway pressure for respiratory distress syndrome | 800 | Drug | Nitric oxide |
| Pratley, Lancet, 2010, 375, 1447 | Inadequate glycaemic control in patients with type 2 diabetes aged 18-80 | 665 | Drug | Liraglutide |
| Ruilope, Lancet, 2010, 375, 1255 | Patients with mild-to-moderate hypertension aged 18-75 | 1328 | Drug | LCZ696 |
| Shakur, Lancet, 2010, 376, 23 | Prevention of death, vascular occlusive events, & receipt of blood transfusion in adult trauma patients | 20211 | Drug | Tranexamic acid |
| Slater, Lancet, 2010, 376, 1225 | Pain-specific brain & spinal cord activity reduction after an acute noxious procedure in newborn infants | 59 | Drug | Sucrose |
| Strang, Lancet, 2010, 375, 1885 | Patients with chronic heroin addiction | 127 | Drug | Heroin (diamorphine/ diacetylmorphine) |
| Weeks, Lancet, 2010, 375, 141 | Women with a retained placenta | 577 | Drug | Saline containing oxytocin |
| Clark, NEJM, 2009, 361, 2424 | Influenza prevention in adults aged 18-50 | 176 | Vaccine | Monovalent influenza A/California/2009 (H1N1) surface-antigen vaccine (MF59-adjuvanted form containing hemagglutinin) |
| Fernandez, NEJM, 2009, 361, 1249 | Patients with untreated acute myeloid leukaemia aged 17-60 | 657 | Drug | Daunorubicin & cytarabine |
| Hurst, NEJM, 2009, 361, 968 | Patients with dupuytren’s disease | 308 | Drug | Collagenase clostridium histolyticum |
| Lassen, NEJM, 2009, 361, 594 | Thromboprophylaxis in patients undergoing total knee replacement | 3195 | Drug | Apixaban |
| Mastronarde, NEJM, 2009, 360, 1487 | Gastroesophageal reflux in patients with poorly controlled asthma | 412 | Drug | Esomeprazole |
| McHutchison, NEJM, 2009, 360, 1827 | Patients with chronic hepatitis C virus genotype 1 infection | 250 | Drug | Telaprevir, peginterferon alfa-2a & ribavirin |
| **First author, journal, year, volume, first page** | **Patient group and intervention target** | **Sample size** | **Intervention type** | **Active ingredients** |
| Mouridsen, NEJM, 2009, 361, 766 | Postmenopausal women with hormone-receptor- positive breast cancer | Unclear | Drug | Letrozole |
| Oviedo-Joekes, NEJM, 2009, 361, 777 | Patients with opioid dependence refractory to treatment | 226 | Drug | Diacetylmorphine |
| Panickar, NEJM, 2009, 360, 329 | Attacks of wheezing induced by upper respiratory viral infections in children aged 10-60 months | 700 | Drug | Prednisolone |
| Smith, NEJM, 2009, 361, 745 | Men with nonmetastatic prostate cancer | 1468 | Drug | Denosumab |
| Taylor, NEJM, 2009, 361, 2113 | Carotid intima-media thickness in patients who had coronary heart disease or a coronary heart disease risk equivalent | 208 | Drug | Extended-release niacin |
| Abdool Karim, NEJM, 2010, 362, 697 | Patients with tuberculosis & HIV | 642 | Drug | Didanosine, lamivudine, & efavirenz (with standard tuberculosis therapy & trimethoprim-sulfamethoxazole) |
| Bashutski, NEJM, 2010, 363, 2396 | Osseous regeneration in patients with severe chronic periodontitis | 40 | Drug | Teriparatide, calcium & vitamin D |
| Chosidow, NEJM, 2010, 362, 896 | Patients with live head lice aged at least 2 | 812 | Drug | Ivermectin |
| Cicardi, NEJM, 2010, 363, 523 | Patients with an acute attack of hereditary angioedema | 72 | Drug | Ecallantide |
| Cummings, NEJM, 2010, 362, 686 | Risk of fractures, breast cancer & cardiovascular disease in women with osteoporosis aged 59-80 | 8556 | Drug | Lasofoxifene |
| Glauser, NEJM, 2010, 362, 790 | Children with newly diagnosed childhood absence epilepsy | 453 | Drug | Ethosuximide |
| Griffiths, NEJM, 2010, 362, 118 | Patients with moderate-to–severe psoriasis | 903 | Drug | Ustekinumab |
| Kantoff, NEJM, 2010, 363, 411 | Men with metastatic castration-resistant prostate cancer | 512 | Vaccine | Sipuleucel-T |
| Kuter, NEJM, 2010, 363, 1889 | Adults with immune thrombocytopenia | 234 | Drug | Romiplostim |
| Lane, NEJM, 2010, 363, 1521 | Pain prevention in patients with osteoarthritis of the knee | 450 | Drug | Tanezumab |

| **First author, journal, year, volume, first page** | **Patient group and intervention target** | **Sample size** | **Intervention type** | **Active ingredients** |
| --- | --- | --- | --- | --- |
| Lassen, NEJM, 2010, 363, 2487 | Thromboprophylaxis in patients undergoing total hip replacement | 5407 | Drug | Apixaban |
| Madhi, NEJM, 2010, 362, 289 | Severe rotavirus gastroenteritis prevention in infants | 4939 | Vaccine | Not provided |
| Martin, NEJM, 2010, 363, 2200 | Women with node-negative breast cancer | 1060 | Drug | Docetaxel, doxorubicin, & cyclophosphamide (TAC) |
| Peters, NEJM, 2010, 363, 1755 | Adults with uncontrolled asthma | 210 | Drug | Tiotropium bromide & glucocorticoid |
| Smith, NEJM, 2010, 363, 245 | Obese/overweight adults | 3182 | Drug | Lorcaserin |
| Sundar, NEJM, 2010, 362, 504 | Patients with visceral leishmaniasis | 412 | Drug | Liposomal amphotericin B |
| Van Der Ploeg, NEJM, 2010, 362, 1396 | Patients with late-onset pompe’s disease aged >=8 | 90 | Drug | Alglucosidase alfa |
| Van Gelder, NEJM, 2010, 362, 1363 | Patients with permanent atrial fibrillation | 614 | Drug | Not provided |
| Zuraw, NEJM, 2010, 363, 513 | Patients with hereditary angioedema | 68 & 22 | Drug | Nanofiltered C1 inhibitor concentrate |
| Connolly, NEJM, 2011, 364, 806 | Stroke prevention in patients with atrial fibrillation | 5599 | Drug | Apixaban |
| Gerstein, NEJM, 2011, 364, 818 | Patients with type 2 diabetes & cardiovascular disease or additional cardiovascular risk factors | Unclear | Drug | Not provided |
| Rosenheck, NEJM, 2011, 364, 842 | Patients with unstable schizophrenia or schizoaffective disorder | 369 | Drug | Risperidone |
| Yao, NEJM, 2011, 364, 514 | Patients with advanced pancreatic neuroendocrine tumours | 410 | Drug | Everolimus |

**Studies evaluating non-pharmacologic interventions**

| **First author, journal, year, volume, first page** | **Patient group and intervention target** | **Sample size** | **Intervention type** | **Active ingredients** |
| --- | --- | --- | --- | --- |
| Ditto, ABM, 2009, 37, 306 | Psychophysiological effects in young adult blood donors | 98 | Behaviour change | Applied tension technique |
| Magnan, ABM, 2009, 37, 46 | Risk and worry perception in smokers | Unclear | Behaviour change | Smoking consequence statements |
| Stock, ABM, 2009, 38, 225 | Sun protection in male outdoor workers | 148 | Behaviour change | UV photo of face & skin cancer educational video |
| Williams, ABM, 2009, 37, 315 | Long-term tobacco abstinence support in adult smokers | 1006 | Behaviour change | Cessation pamphlets & information on local treatment programs |
| Hendershot, ABM, 2010, 40, 77 | Alcohol-related health risk reduction in young adults made aware of their ALDH2 genotype | 200 | Behaviour change | Health risk information specific to alcohol-related cancer/ alcohol dependence depending on ALDH2 genotype |
| Lawler, ABM, 2010, 39, 250 | Patients with type 2 diabetes or hypertension | 434 | Behaviour change | Not specified |
| Adamsen, BMJ, 2009, 339, 895 | Patients with cancer undergoing chemotherapy or treatment for advanced disease | 269 | Behaviour change | Not specified |
| Boysen, BMJ, 2009, 339, 273 | Physical activity improvement in patients with ischaemic stroke aged >= 40 | 314 | Behaviour change | Repeated instructions about physical activity |
| Custers, BMJ, 2009, 339, 1065 | Women having intrauterine insemination | 391 | Body positioning | Immobilisation after insemination |
| Dennis, BMJ, 2009, 338, 280 | Postnatal depression prevention in high-risk women | 701 | Behaviour change | Not specified |
| Engebretsen, BMJ, 2009, 339, 729 | Patients with subacromial shoulder pain | 104 | Shockwave therapy/ rehabilitation | Radial extracorporeal shockwave treatment |
| Goud, BMJ, 2009, 338, 1132 | Decision making in cardiac rehabilitation teams | Unclear | Behaviour change | Not specified |

| **First author, journal, year, volume, first page** | **Patient group and intervention target** | **Sample size** | **Intervention type** | **Active ingredients** |
| --- | --- | --- | --- | --- |
| Hoff, BMJ, 2009, 338, 1363 | Colorectal cancer screening in adults aged 55-64 | 55736 | Screening | Flexible sigmoidoscopy with/without faecal occult blood testing |
| Hupperets, BMJ, 2009, 339, 276 | Ankle sprain recurrence in athletes aged 12-70 | 522 | Rehabilitation | Not specified |
| Larsen, BMJ, 2009, 338, 1253 | First & second year registrars specialising in gynaecology & obstetrics undertaking laparoscopic operations | 24 | Simulator training | Not specified |
| Lawton, BMJ, 2009, 338, 88 | Women not undertaking 30 minutes of moderate intensity physical activity at least five days a week aged 40-74 | 1089 | Behaviour change | Exercise prescription |
| Little, BMJ, 2009, 339, 329 | Women with a cytology result showing borderline nuclear abnormalities or mild dyskaryosis aged 20-59 | 4439 | Screening | Cytological screening |
| Martins, BMJ, 2009, 339, 1131 | Treatment completion in adults with tuberculosis aged >=18 | 270 | Food supplementation | Food incentives: meal & food package |
| Ndekha, BMJ, 2009, 338, b1867 | Wasted adults with HIV who were starting antiretroviral therapy with BMI <18.5 | 491 | Food supplementation | Food supplement: ready-to-use fortified spread |
| O'Sullivan, BMJ, 2009, 338, 880 | Obstetric & neonatal outcomes in nulliparous non-diabetic women at term | 2426 | Food supplementation | Not specified |
| Ravaud, BMJ, 2009, 338, b421 | Patients with osteoarthritis of the knee | 336 | Behaviour change | Education on osteoarthritis & treatment management, information on physical exercises, information on weight loss |
| Sackley, BMJ, 2009, 339, 670 | Mobility & activity limitations in care home residents | 249 | Rehabilitation | Not specified |
| Van Linschoten, BMJ, 2009, 339, 1010 | Patients with patellofemoral pain syndrome | 131 | Behaviour change | Supervised exercise, information about patellofemoral pain syndrome, instructions for home exercises |
| Volandes, BMJ, 2009, 338, 1372 | Advanced care planning in patients with dementia aged >=65 | 200 | Behaviour change | Depiction of patient with advanced dementia & verbal description of condition |
| **First author, journal, year, volume, first page** | **Patient group and intervention target** | **Sample size** | **Intervention type** | **Active ingredients** |
| Wake, BMJ, 2009, 339, 1132 | Overweight/mild obesity in children aged 5-10 | 258 | Behaviour change | Not specified |
| Young, BMJ, 2009, 339, 899 | Consent for organ donation in relatives of prospective donors | 201 | Behaviour change | Request for organ donation consent by potential donor’s clinician & a donor transplant coordinator (organ procurement officer) |
| Ansah, BMJ, 2010, 340, 930 | Malaria diagnosis in patients with suspected Malaria | 7263 | Diagnostic testing | Not specified |
| Banerjee, BMJ, 2010, 340, 1291 | Immunisation rates in children aged 1-3 | 1640 | Behaviour change | Incentives: raw lentils & metal plates provided for completed immunisation |
| Barrowclough, BMJ, 2010, 341, 1204 | Patients with psychosis & comorbid substance use problem | 327 | Behaviour change | Integrated motivational interviewing & cognitive behavioural therapy |
| Bennell, BMJ, 2010, 341, 82 | Patients with chronic rotator cuff disease | 120 | Rehabilitation | Standardised manual therapy |
| Bleakley, BMJ, 2010, 340, 1122 | Ankle function in patients with acute ankle sprains | 101 | Rehabilitation | Not specified |
| Detering, BMJ, 2010, 340, 847 | End of life care in elderly patients aged >=80 | 309 | Behaviour change | Facilitated advance care planning |
| Dormandy, BMJ, 2010, 341, 926 | Sickle cell disease & thalassaemia screening in pregnant women | 1708 | Screening | Tests for sickle cell disease & thalassaemia offered to both parents when pregnancy first reported |
| Duran-Cantolla, BMJ, 2010, 341, 1142 | Systemic hypertension in patients with obstructive sleep apnoea | 340 | Device | Continuous positive airway pressure |
| Ford, BMJ, 2010, 340, 250 | Obese young people aged 9-17 | 106 | Behaviour change | Providing real time feedback |
| Haran, BMJ, 2010, 340, 1345 | Fall prevention in older, regular wearers of multifocal glasses | 606 | Device | Single lens distance glasses |
| Jafar, BMJ, 2010, 341, 29 | Blood pressure in children and adults aged 5-39 | 4023 | Behaviour change | Family focussed home health education |

| **First author, journal, year, volume, first page** | **Patient group and intervention target** | **Sample size** | **Intervention type** | **Active ingredients** |
| --- | --- | --- | --- | --- |
| Lambeek, BMJ, 2010, 340, 750 | Chronic low back pain in adults aged 18-65 | 134 | Difficult to specify | Not specified |
| Marteau, BMJ, 2010, 340, 1176 | Screening uptake in adults aged 40-69 at risk for diabetes | 1272 | Behaviour change | Invitation describing diabetes as a serious potential problem, details of possible costs & benefits of screening & treatment |
| Nourhashemi, BMJ, 2010, 340, 1346 | Patients with mild to moderate Alzheimer’s disease | 1131 | Difficult to specify | Specific care plan: standardised twice yearly consultation, guidelines for management |
| Van Peperstraten, BMJ, 2010, 341, 712 | Decision making in couples (women aged <40) on waiting list for first in vitro fertilisation cycle | 308 | Behaviour change | Support of nurse specialist & offer of reimbursement (extra treatment cycle) |
| Wearden, BMJ, 2010, 340, 959 | Patients with chronic fatigue syndrome/myalgic encephalomyelitis or encephalitis aged >=18 | 296 | Behaviour change | Not specified |
| Boers, BMJ, 2011, 342, 35 | Pregnant women with suspected intrauterine growth restriction near term | 650 | Labour induction | Induction of labour |
| Hostler, BMJ, 2011, 342, 371 | Cardiopulmonary resuscitation of patients having cardiac arrest outside hospital | 1586 | Behaviour change | Real-time feedback on cardiopulmonary resuscitation |
| Smith, BMJ, 2011, 342, 482 | Patients with type 2 diabetes | 395 | Behaviour change | Not specified |
| Bakitas, JAMA, 2009, 302, 741 | Patients with advanced cancer (gastrointestinal tract, lung, breast) | 322 | Behaviour change | Not specified |
| Flynn, JAMA, 2009, 301, 1451 | Medically stable patients with heart failure | 2331 | Behaviour change | Not specified |
| McDermott, JAMA, 2009, 301, 165 | Patients with peripheral arterial disease with or without intermittent claudication | 156 | Behaviour change | Not specified |
| Montalescot, JAMA, 2009, 302, 947 | Patients with acute coronary syndromes | 352 | Device | Angioplasty |
| Morey, JAMA, 2009, 301, 1883 | Overweight survivors of colorectal, breast, and prostate cancer aged 65-91 | 641 | Behaviour change | Not specified |

| **First author, journal, year, volume, first page** | **Patient group and intervention target** | **Sample size** | **Intervention type** | **Active ingredients** |
| --- | --- | --- | --- | --- |
| Stanley, JAMA, 2009, 301, 1460 | Patients with late-life generalized anxiety disorder | 134 | Behaviour change | Cognitive behaviour therapy,education, motivational interviewing |
| Taccone, JAMA, 2009, 302, 1977 | Adult patients with moderate or severe hypoxemia & acute respiratory distress syndrome receiving mechanical ventilation | 342 | Body positioning | Prone positioning |
| Timsit, JAMA, 2009, 301, 1231 | Major catheter-related infection prevention in adults aged >=18 | 2095 | Device | Chlorhexidine gluconate-impregnated sponge & scheduled change of unsoiled adherent dressings |
| Young, JAMA, 2009, 301, 1547 | Coronary artery disease screening in patients with type 2 diabetes | 1123 | Screening | Adenosine-stress radionuclide myocardial perfusion imaging |
| Annema, JAMA, 2010, 304, 2245 | Patients with resectable (suspected) non-small cell lung cancer | 241 | Surgical | Mediastinoscopy, & thoracotomy with lymph node dissection when no evidence of mediastinal tumor spread |
| Bennett-Guerrero, JAMA, 2010, 304, 755 | Sternal wound infection prevention in cardiac surgical patients at high risk (diabetes, body mass index >30, or both) | 1502 | Surgical | Gentamicin-collagen sponges |
| Church, JAMA, 2010, 304, 2253 | Haemoglobin A1c in sedentary adults with type 2 diabetes | 262 | Behaviour change | Not specified |
| Dykes, JAMA, 2010, 304, 1912 | Fall prevention in acute care units | 10264 | Difficult to specify | Posters, patient education handouts, plans of care (all communicated patient-specific alerts) |
| Hajjar, JAMA, 2010, 304, 1559 | Blood transfusion in adult patients undergoing cardiac surgery with cardiopulmonary bypass | 502 | Transfusion | Red blood cells |
| Piacentini, JAMA, 2010, 303, 1929 | Tourette or chronic tic disorder in children aged 9-17 | 126 | Behaviour change | Behaviour therapy |
| Powell, JAMA, 2010, 304, 1331 | Patients with mild to moderate heart failure | 902 | Behaviour change | Not specified |
| **First author, journal, year, volume, first page** | **Patient group and intervention target** | **Sample size** | **Intervention type** | **Active ingredients** |
| Safren, JAMA, 2010, 304, 875 | Adults with attention-deficit/hyperactivity disorder | 86 | Behaviour change | Cognitive behavioural therapy |
| Tiwari, JAMA, 2010, 304, 536 | Depressive symptoms in women aged>=18 with a history of intimate partner violence | 200 | Behaviour change | Not specified |
| Giuliano, JAMA, 2011, 305, 569 | Women with invasive breast cancer | 891 | Surgical | Lumpectomy & tangential whole-breast irradiation, sentinel lymph node dissection, & complete axillary lymph node dissection (ALND) of 10 or more nodes |
| Goldman, JAMA, 2011, 305, 167 | Patients undergoing coronary artery bypass grafting | 757 | Surgical | Radial artery graft |
| Scales, JAMA, 2011, 305, 363 | Delivery of 6 evidence-based practices in intensive care units | Unclear | Behaviour change | Not specified |
| Arifeen, Lancet, 2009, 374, 393 | Health and nutrition in children aged <5 | Unclear | Difficult to specify | Not specified |
| Hall, Lancet, 2009, 373, 390 | Infants with pyloric stenosis | 180 | Surgical | Laparoscopic pyloromyotomy |
| Jarvik, Lancet, 2009, 374, 1074 | Patients with carpal tunnel syndrome without denervation | 116 | Surgery/non-surgical therapy | Not specified |
| Kerac, Lancet, 2009, 374, 136 | Severe acute malnutrition in children aged 5-168 months | 795 | Food supplementation | Synbiotic2000 Forte |
| Santarius, Lancet, 2009, 374, 1067 | Patients with chronic subdural haematoma requiring burr-hole drainage aged >=18 | 215 | Surgical | Drain |
| Schweickert, Lancet, 2009, 373, 1874 | Functional outcomes in sedated adults in intensive care unit on mechanical ventilation >=18 years | 104 | Rehabilitation | Mobilisation & daily interruption of sedation |
| Sebag-Montefiore, Lancet, 2009, 373, 811 | Patients with rectal cancer | 1350 | Radiotherapy | Preoperative radiotherapy |
| Corbett, Lancet, 2010, 376, 1244 | Smear-positive tuberculosis diagnosis in adults aged >=16 | 110432 | Diagnostic testing | Sputum specimens assessed by fluorescence microscopy |

| **First author, journal, year, volume, first page** | **Patient group and intervention target** | **Sample size** | **Intervention type** | **Active ingredients** |
| --- | --- | --- | --- | --- |
| Kuck, Lancet, 2010, 375, 31 | Stable ventricular tachycardia in patients with coronary heart disease aged 18-80 | Unclear | Surgical | Catheter ablation before implantation of cardioverter defibrillator |
| Nout, Lancet, 2010, 375, 816 | Patients with stage I or IIA endometrial carcinoma with features of high-intermediate risk | 427 | Radiotherapy | Vaginal brachytherapy |
| Andriole, NEJM, 2009, 360, 1310 | Prostate cancer screening in men | 76693 | Screening | Screening with prostate-specific-antigen testing & digital rectal examination |
| Azzopardi, NEJM, 2009, 361, 1349 | Infants with perinatal asphyxial encephalopathy aged <6 hours | 325 | Body cooling | Cooling of the body to 33.5 degrees Celsius & intensive care |
| Bellomo, NEJM, 2009, 361, 1627 | Critically ill adults with acute kidney injury | 1508 | Renal replacement therapy | Postdilution continuous venovenous hemodiafiltration |
| Cantor, NEJM, 2009, 360, 2705 | Patients with myocardial infarction | 1059 | Device | Percutaneous coronary intervention (with aspirin, tenecteplase, & heparin/ enoxaparin; concomitant clopidogrel recommended) |
| Green, NEJM, 2009, 361, 245 | Anxiety, depression, & test-related distress in asymptomatic adults who had a parent with Alzheimer’s disease | 162 | Behaviour change | Receive results of personal apolipoprotein E (APOE) genotype |
| Landon, NEJM, 2009, 361, 1339 | Women with mild gestational diabetes mellitus | 958 | Behaviour change | Not specified |
| Moers, NEJM, 2009, 360, 7 | Preservation of deceased donor kidneys for transplantation | 672 | Organ preservation | Hypothermic machine perfusion of kidney |
| Subak, NEJM, 2009, 360, 481 | Urinary incontinence in overweight & obese women | 338 | Behaviour change | Not specified |
| Tonino, NEJM, 2009, 360, 213 | Patients with multivessel coronary artery disease receiving percutaneous coronary intervention | 1005 | Device | Percutaneous coronary intervention & implantation of drug-eluting stents |

| **First author, journal, year, volume, first page** | **Patient group and intervention target** | **Sample size** | **Intervention type** | **Active ingredients** |
| --- | --- | --- | --- | --- |
| Bennett-Guerrero, NEJM, 2010, 363, 1038 | Surgical-site infection prevention in patients undergoing colorectal surgery | 602 | Surgical | Gentamicin-collagen sponges (& standard care, including prophylactic systemic antibiotics) |
| Cooper, NEJM, 2010, 363, 609 | Progressive chronic kidney disease in patients aged >=18 | 828 | Renal replacement therapy | Dialysis |
| Frobell, NEJM, 2010, 363, 331 | Torn anterior cruciate ligament of the knee in young active adults | 121 | Surgery/ rehabilitation | Anterior cruciate ligament reconstruction |
| Garcia-Pagan, NEJM, 2010, 362, 2370 | Patients with cirrhosis & acute variceal bleeding | 63 | Device | Transjugular intrahepatic portosystemic shunt treatment with a polytetrafluoroethylene-covered stent |
| Greenhalgh, NEJM, 2010, 362, 1872 | Patients with large abdominal aortic aneurysms (>=5.5 cm in diameter) physically ineligible for open repair | 404 | Surgical | Endovascular repair |
| Grobbee, NEJM, 2010, 362, 1881 | Patients with an abdominal aortic aneurysm of at least 5cm in diameter | 351 | Surgical | Open repair |
| Haskal, NEJM, 2010, 362, 494 | Patients with venous anastomotic stenosis in failing haemodialysis grafts | 190 | Device | Conventional balloon angioplasty & expanded polytetrafluoroethylene endovascular stent graft |
| Katon, NEJM, 2010, 363, 2611 | Patients with depression & poorly controlled diabetes, &/or coronary heart disease | 214 | Difficult to specify | Not specified |
| Knip, NEJM, 2010, 363, 1900 | Beta-cell autoimmunity in infants with HLA-conferred susceptibility to type 1 diabetes & at least one family member with type 1 diabetes | 230 | Food supplementation | Casein hydrolysate formula |
| Serruys, NEJM, 2010, 363, 136 | Patients requiring treatment with coronary stents | 2292 | Device | Coronary stents releasing zotarolimus |
| Wennberg, NEJM, 2010, 363, 1245 | Medical costs & resource utilization for patients with selected medical conditions & predicted high health care costs | 174120 | Behaviour change | Not specified |

Studies evaluating pharmacologic and non-pharmacologic interventions

| **First author, journal, year, volume, first page** | **Patient group and intervention target** | **Sample size** | **Intervention type** | **Active ingredients** |
| --- | --- | --- | --- | --- |
| Morin, JAMA, 2009, 301, 2005 | Adults with persistent insomnia | 160 | Behaviour change/drug | Cognitive behavioural therapy & zolpidem |
| Widmark, Lancet, 2009, 373, 301 | Patients with locally advanced prostate cancer | 875 | Drug/radiotherapy | Flutamide |
| Patel, Lancet, 2010, 376, 2086 | Adults with depression & anxiety disorders | 2796 | Difficult to specify | Not specified |
| Mintz-Hittner, NEJM, 2011, 364, 603 | Patients with zone I or zone II posterior stage 3+ retinopathy of prematurity | 150 | Drug/laser therapy | Bevacizumab |
